# Pins, Needles, and a Pounding Heart: A Post-Lap-Band Surprise

**DOI:** 10.7759/cureus.107551

**Published:** 2026-04-22

**Authors:** Anne Y Chua, Maxwell Bressman

**Affiliations:** 1 Internal Medicine, Greenwich Hospital, Yale New Haven Health, Greenwich, USA

**Keywords:** adjustable gastric band complications, laparoscopic adjustable gastric band, paresthesia, sinus tachycardia, vagal nerve irritation

## Abstract

Neurologic and cardiovascular symptoms are uncommon complications of laparoscopic adjustable gastric banding (LAGB). Alterations in vagal tone have been reported, most commonly as vasovagal syncope or bradycardia. We describe a unique presentation of sinus tachycardia and paresthesias resulting from vagal nerve irritation due to gastric band adjustments.

A 51-year-old woman with a past medical history of rheumatoid arthritis and laparoscopic adjustable gastric band surgery presented with episodic right-sided paresthesias, palpitations, and sinus tachycardia occurring predominantly during and after meals. Vital signs were normal, and initial blood tests were unremarkable. Telemetry revealed periods of sinus tachycardia up to 160 beats per minute. Extensive neurologic, infectious, and cardiopulmonary evaluation was unrevealing. Further history revealed similar symptoms following prior gastric band adjustment. After removing 4.2 mL of fluid from the gastric band, the patient had complete resolution of symptoms.

Surgical placement of an adjustable gastric band is close to the anterior vagal trunk, which courses near the gastroesophageal junction. Placement or adjustments of the gastric band can irritate or compress the vagal nerve, leading to unintended autonomic responses. Even without symptoms at rest, expansion of the stomach from food can lead to a similar response. While vagal nerve stimulation normally produces bradycardia, this case demonstrates that vagal dysfunction can present with paradoxical sinus tachycardia and sensory symptoms, all of which resolved with decreased gastric band pressure.

In patients with prior adjustable gastric banding, vagal nerve stimulation should be considered for unexplained autonomic or neurologic symptoms. Early recognition can prevent unnecessary testing and facilitate prompt management. This case highlights the importance of recognizing neurologic and cardiovascular complications of gastric banding to provide appropriate management.

## Introduction

Laparoscopic adjustable gastric banding (LAGB) was once a routine bariatric procedure for obesity. With the advent of effective medical therapy, including GLP-1 agonists, and newer surgical approaches, LAGB has declined in prevalence [1]. Long-term complications of LAGB are well-described, including band slippage, erosion, and obstruction [2]. Neurologic and autonomic cardiovascular complications are atypical. The adjustable gastric band is positioned laparoscopically around the proximal stomach, just below the gastroesophageal junction, an area closely associated with the anterior and posterior vagal trunks, making the vagal nerve susceptible to mechanical irritation depending on the band placement.

Vagal nerve involvement related to gastric banding has been reported and typically presents as vasovagal syncope or bradycardia [3]. Isolated sinus tachycardia and paresthesias due to vagal nerve irritation have not been described in the literature. We present a case of sinus tachycardia and paresthesias resulting from over-adjustment of a gastric band.

## Case presentation

**Figure 1:**
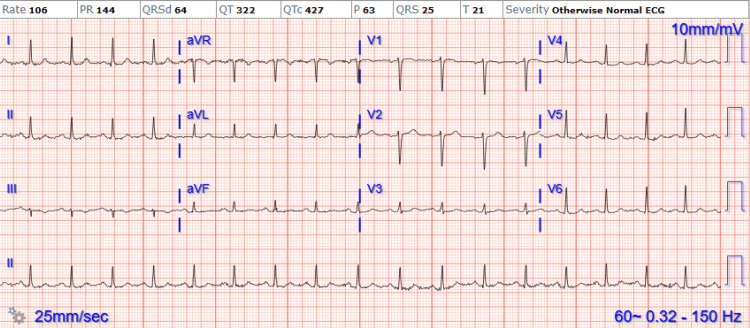
ECG Showing Sinus Tachycardia ECG: Electrocardiogram

A 51-year-old female with a history of rheumatoid arthritis and laparoscopic adjustable gastric band placement 10 years prior presented with episodic right-sided paresthesias in the upper extremity, palpitations, and tachycardia that were prandial and post-prandial. She described the paresthesias as transient tingling and numbness in her right arm. She had no other symptoms, and review of systems was negative. She had never experienced paresthesias in the past and had no report of headaches on this admission or previously. Vital signs were normal on admission except for intermittent regular tachycardia, with heart rates ranging from 90 to 160 beats per minute during and after eating and with ambulation. Physical examination was unremarkable, with electrocardiogram (ECG) (Figure 1) and telemetry showing the tachycardia to be sinus rhythm.

**Figure 2:**
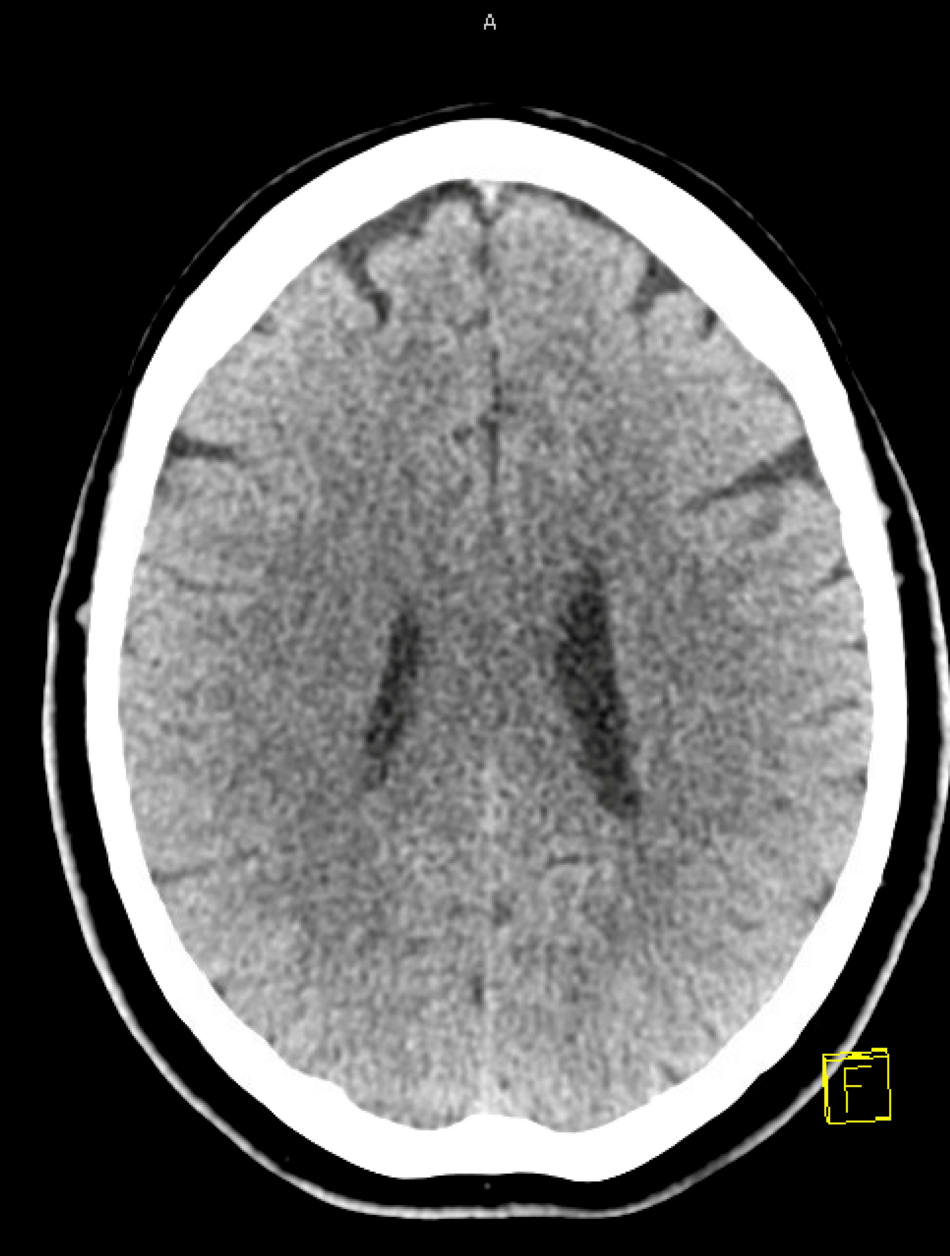
Normal CT Head CT: Computed Tomography

**Figure 3:**
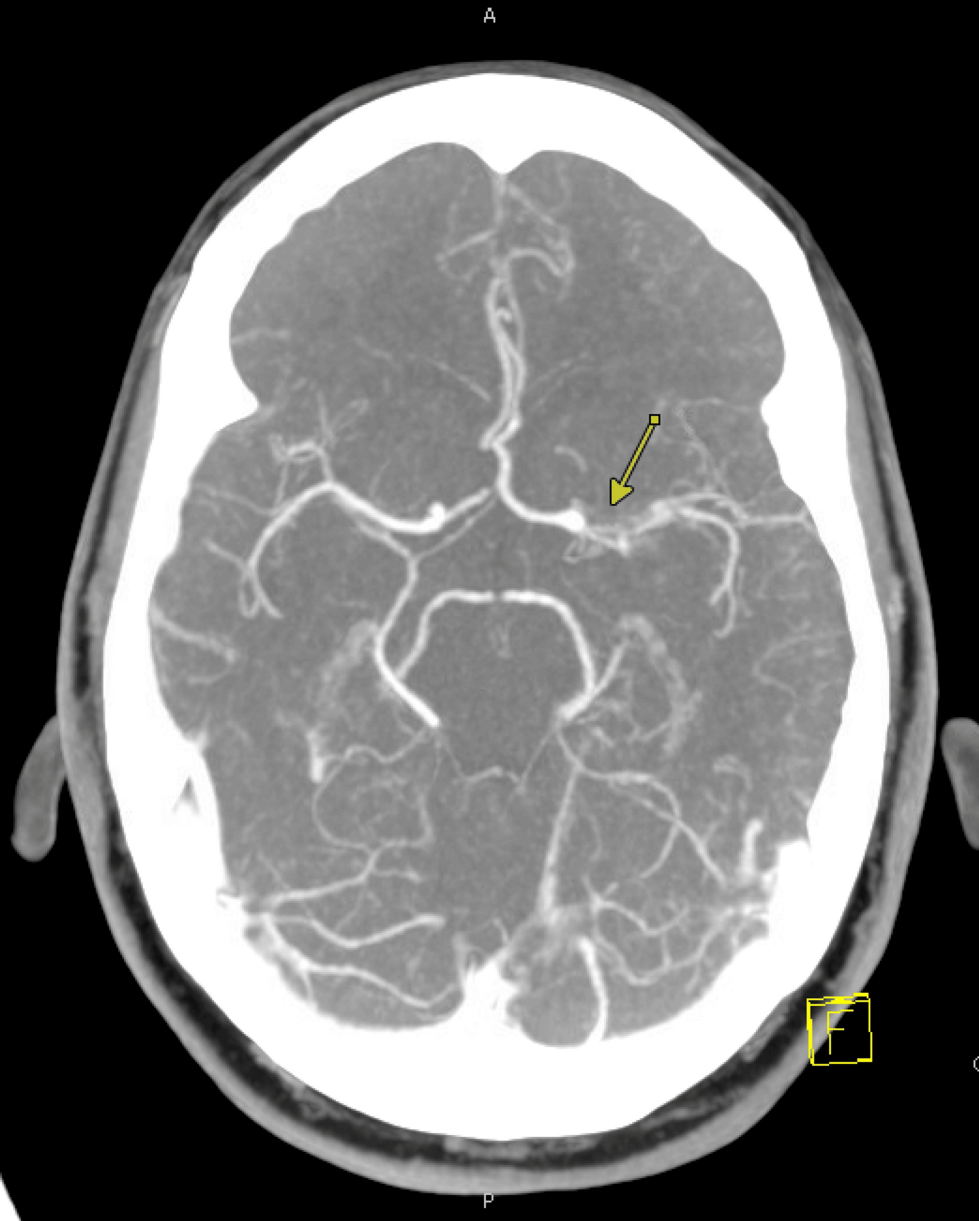
CTA Head With a Chronically Occluded Left MCA as Demonstrated by the Yellow Arrow CTA: Computed Tomography Angiography; MAC: Middle Cerebral Artery

**Figure 4:**
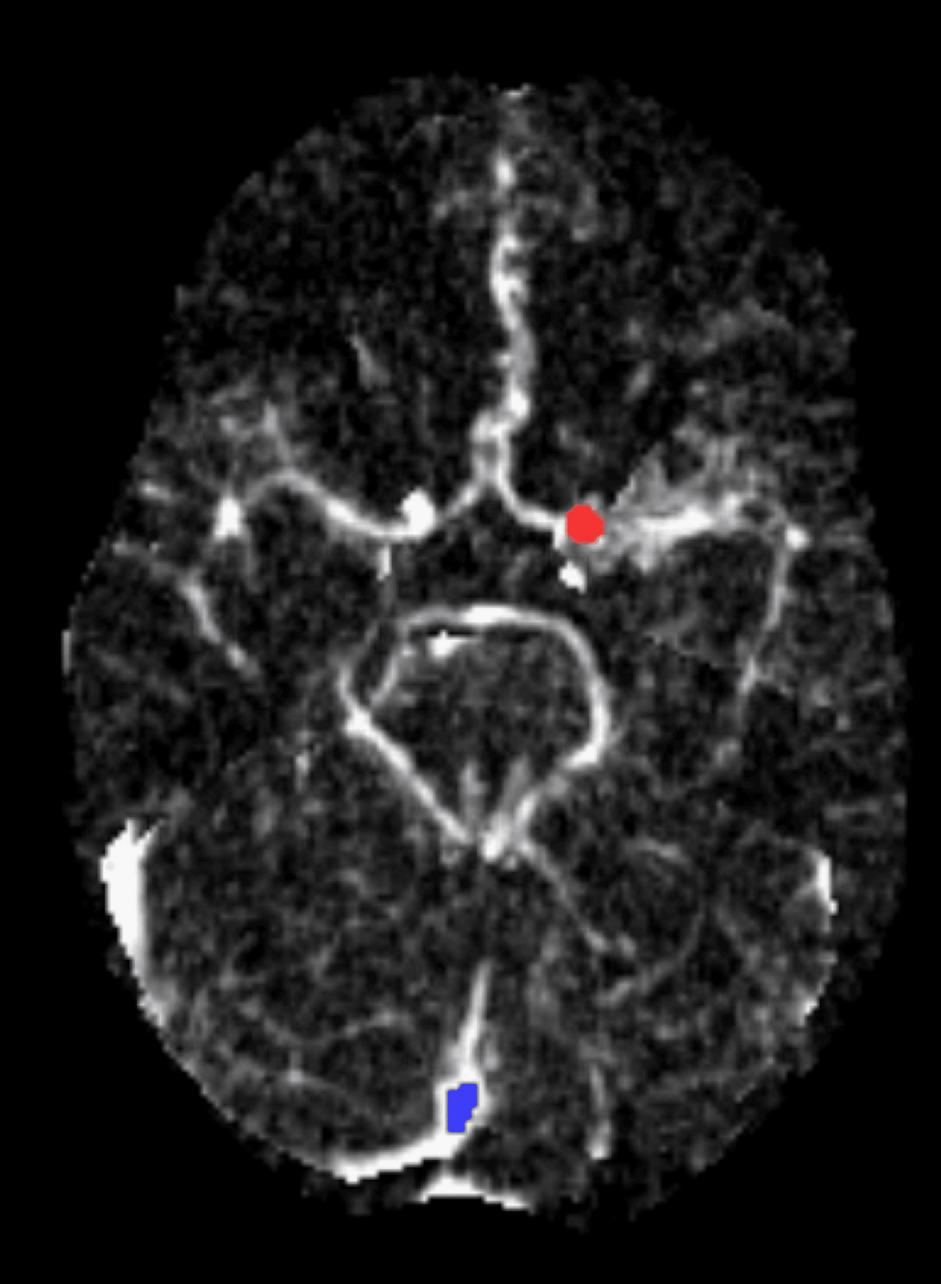
Normal CT Perfusion Normal CT perfusion with the red and blue dots showing the contrast phases performed for a neuroperfusion study. CT: Computed Tomography

**Figure 5:**
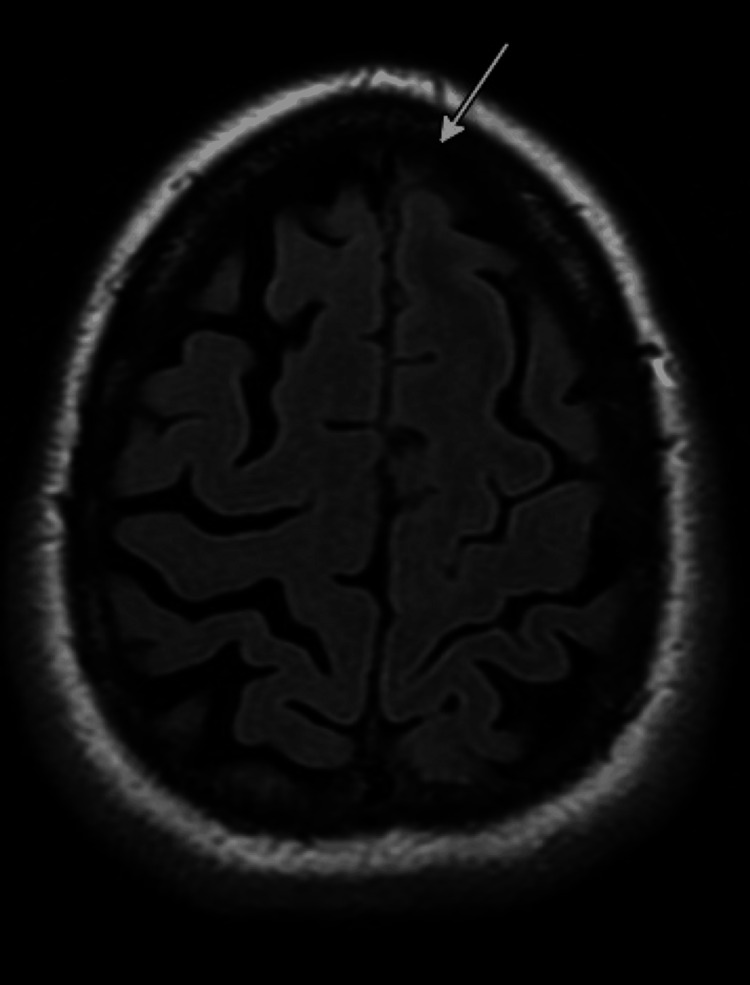
MRI Brain With Arrow Showing Left Meningeal Enhancement MRI: Magnetic Resonance Imaging

**Figure 6:**
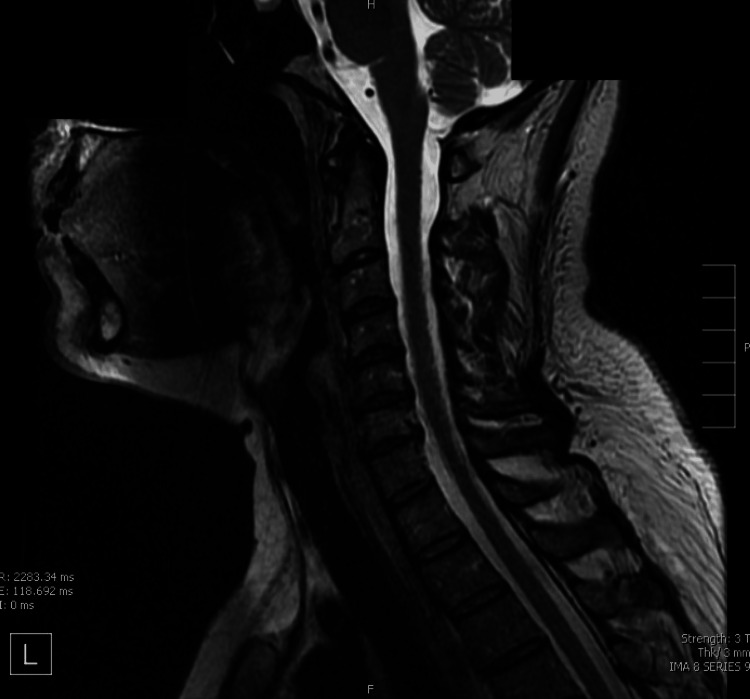
Normal MRI of the Cervical Spine MRI: Magnetic Resonance Imaging

Initial blood tests showed normal blood counts, normal kidney function, and electrolytes. Computed tomography (CT) head (Figure 2), computed tomography angiography (CTA) of the head and neck (Figure 3), and CT neuroperfusion (Figure 4) were followed by magnetic resonance imaging (MRI) of the brain and cervical spine (Figures 5-6). Imaging showed a chronically occluded left middle cerebral artery and asymmetric convexity meningeal enhancement, greater on the left than on the right. Lumbar puncture was then performed, which revealed three red blood cells, a total nucleated cell count of 2 cells/µL with lymphocytic predominance (89% lymphocytes), glucose of 50 mg/dL, and protein of 39 mg/dL. Cytology showed atypical cerebrospinal fluid with a mononuclear-predominant inflammatory infiltrate.

The patient was empirically treated with acyclovir and ampicillin for suspected meningitis. Workup, including cryptococcal antigen, Lyme disease, West Nile virus, enterovirus, herpes simplex virus, varicella zoster virus, and *Listeria monocytogenes*, was negative, and antimicrobials were discontinued. CT of the chest, abdomen, and pelvis showed no evidence of malignancy or acute pathology.

**Figure 7:**
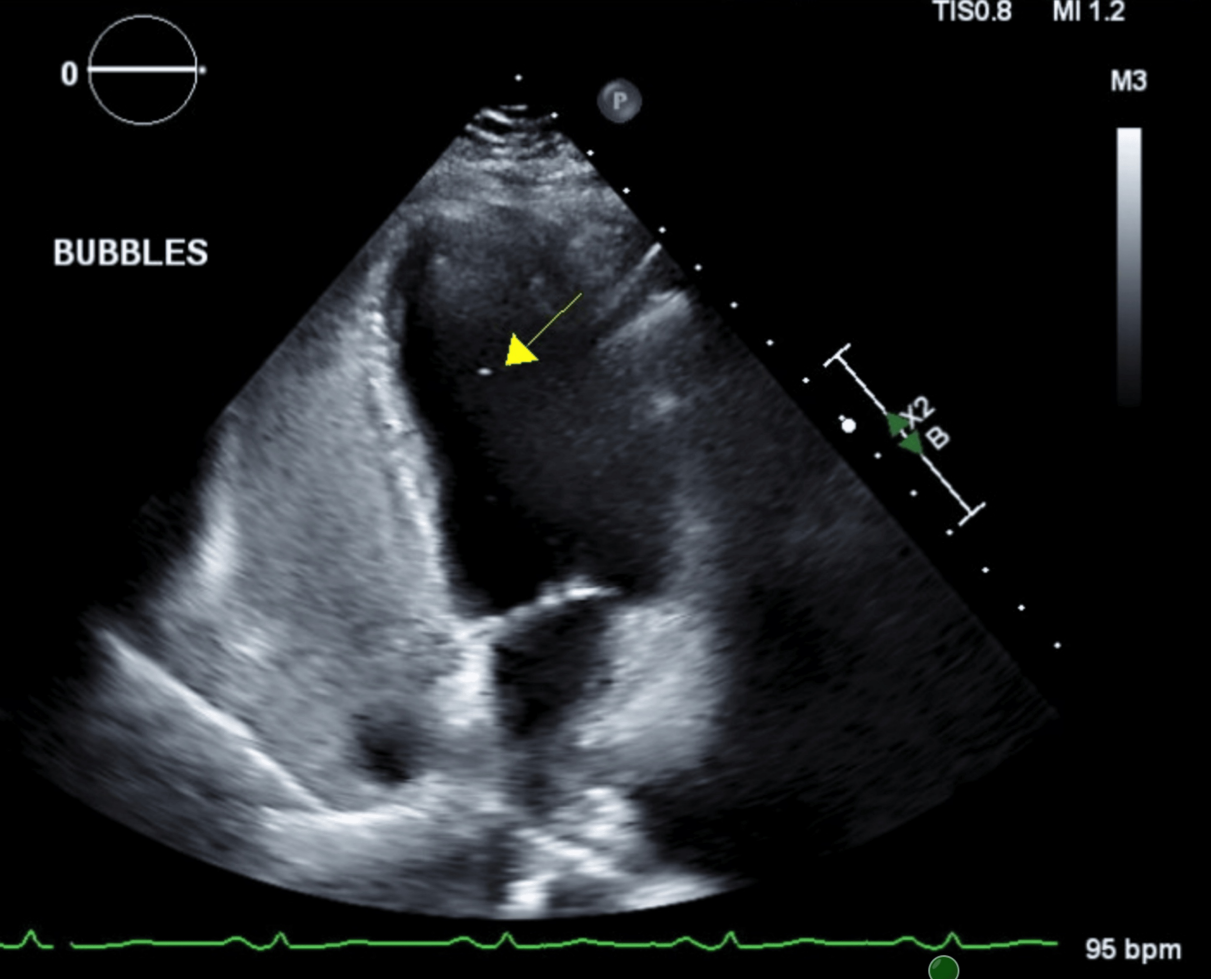
TTE With Bubble Study Normal TTE with a possible patent foramen ovale with a bubble present in the left ventricle, shown by the yellow arrow. TTE: Transthoracic Echocardiogram

The patient’s paresthesias resolved spontaneously; however, palpitations and sinus tachycardia persisted. Telemetry continued to show sinus tachycardia revolving around food and liquid consumption, up to 160 beats per minute. Transthoracic echocardiogram revealed normal cardiac structure and function with a possible patent foramen ovale, which was not felt to be contributory (Figure 7).

The patient disclosed that she had experienced similar episodes of heart rate variability and presyncope with prior gastric band fluid adjustments. She did not associate these symptoms of fluctuation in heart rates with adjustments to her gastric band in the past; however, in retrospect, she was able to provide that detail specifically regarding her heart rates and presyncope. She had never experienced paresthesia in the past, however. Bariatric surgery was consulted, and 4.2 mL of fluid was removed from the gastric band. Following band deflation, the patient experienced immediate and complete resolution of tachycardia and palpitations, with no recurrence on follow-up.

## Discussion

This case highlights an uncommon autonomic complication of LAGB related to vagal nerve irritation. The anterior vagal trunk courses along the lesser curvature of the stomach near the gastroesophageal junction, an area directly adjacent to the typical placement of an adjustable gastric band [4,5]. Mechanical compression, traction, or irritation of the vagal nerve in this region may lead to dysregulated signaling.

Vagal nerve involvement following gastric banding has previously been reported, most commonly presenting as vasovagal syncope or bradycardia. Sedrak et al. described recurrent vasovagal syncopal episodes in patients with adjustable gastric bands, which resolved following band removal or conversion to sleeve gastrectomy [3]. However, paradoxical manifestations such as isolated sinus tachycardia and sensory disturbances, as seen in this patient, are rarely reported and may represent an underrecognized form of vagal dysfunction rather than classic vagal overactivity. More specifically, in this case, the chronically occluded left MCA could also have been a confounding factor, adding to the compromised neurological disturbances with right-sided paresthesias.

Autonomic imbalance resulting from vagal nerve irritation may produce differing cardiovascular responses depending on the degree and nature of nerve involvement. Disruption of vagal afferent signaling may lead to compensatory sympathetic activation, resulting in sinus tachycardia rather than bradycardia, though this has not been widely reported. Symptoms were reproducible with meals and ambulation and completely resolved following gastric band deflation, strongly supporting a mechanical and neurogenic etiology rather than a primary cardiac or neurologic disorder [6]. 

LAGB is known to cause a variety of long-term complications, most commonly including band slippage, pouch dilation, esophageal dysmotility, gastroesophageal reflux disease, and band erosion [2,7-10]. These recognized complications further support the plausibility of vagal nerve involvement in patients presenting with unexplained autonomic symptoms following gastric banding. Long-term follow-up studies and systematic review data further demonstrate substantial complication and reoperation rates after adjustable gastric banding, underscoring the continued relevance of late band-related presentations in current practice [1,2].

The nonspecific presentation in this case led to extensive diagnostic evaluation, including neuroimaging, cerebrospinal fluid analysis, and cardiac testing. Understanding the anatomical relationship between the gastric band and vagal nerve could have enabled clinicians to defer expensive and invasive testing and pursue a trial of fluid removal first [4,5,8]. Furthermore, detailed history taking could have led to earlier pattern recognition in identifying the root cause of her symptoms and avoiding extensive investigations. Multidisciplinary evaluation with a bariatric surgeon is appropriate in such cases, with a simple mechanical adjustment ultimately being the necessary treatment. Improvement after band adjustment or removal has also been reported in other band-related functional complications, further supporting this approach [3,10].

Although the use of adjustable gastric bands has declined in recent years, many patients continue to live with these devices years after implantation [1,2]. Physicians should remain aware of both common and uncommon late complications. Recognition of vagal nerve irritation as a potential cause of unexplained autonomic or neurologic symptoms may prevent unnecessary diagnostic testing and facilitate prompt symptom resolution.

## Conclusions

Adjustable gastric banding can rarely result in vagal nerve irritation, causing atypical autonomic manifestations, including sinus tachycardia and paresthesias. Physicians should consider this etiology in patients with unexplained autonomic or neurologic symptoms and a history of gastric banding. Early recognition allows for simple intervention with rapid symptom resolution and avoidance of unnecessary diagnostic testing.

## References

[1] (2026). Types of weight-loss surgery. https://www.niddk.nih.gov/health-information/weight-management/bariatric-surgery/types.

[2] Shen X, Zhang X, Bi J, Yin K (2015). Long-term complications requiring reoperations after laparoscopic adjustable gastric banding: a systematic review. Surg Obes Relat Dis.

[3] Sedrak MF, Holewijn RA, Horgan S (2012). Gastric band removal and conversion to sleeve gastrectomy for vasovagal syncopal episodes associated with adjustable gastric banding placed using pars flaccida technique. Surg Obes Relat Dis.

[4] Powley TL, Baronowsky EA, Gilbert JM (2013). Vagal afferent innervation of the lower esophageal sphincter. Auton Neurosci.

[5] Baquiran M, Hwang K, Waseem M (2023). Anatomy, head and neck: anterior vagus nerve. StatPearls [Internet].

[6] van Orshoven NP, Oey PL, van Schelven LJ, Roelofs JM, Jansen PA, Akkermans LM (2004). Effect of gastric distension on cardiovascular parameters: gastrovascular reflex is attenuated in the elderly. J Physiol.

[7] Kirshtein B, Lantsberg L, Mizrahi S, Avinoach E (2010). Bariatric emergencies for non-bariatric surgeons: complications of laparoscopic gastric banding. Obes Surg.

[8] Naef M, Mouton WG, Naef U, van der Weg B, Maddern GJ, Wagner HE (2011). Esophageal dysmotility disorders after laparoscopic gastric banding - an underestimated complication. Ann Surg.

[9] Eid I, Birch DW, Sharma AM, Sherman V, Karmali S (2011). Complications associated with adjustable gastric banding for morbid obesity: a surgeon's guides. Can J Surg.

[10] Rogers AM (2010). Improvement of esophageal dysmotility after conversion from gastric banding to gastric bypass. Surg Obes Relat Dis.

